# Supplementary material for: HIVprotI: an integrated web based platform for prediction and design of HIV proteins inhibitors
Source: J Cheminform. 2018 Mar 9;10:12. doi: 10.1186/s13321-018-0266-y (PMC5845081; doi:10.1186/s13321-018-0266-y)
Supplement: Supplementary file 2 — Additional file 2. Source code of HIVProtI web server. [file 13321_2018_266_MOESM2_ESM.zip › HIVprotI_Source-code/hivprotI/weka-3-6-6/doc/weka/classifiers/bayes/package-summary.html]

weka.classifiers.bayes


---


|  |  |  |
| --- | --- | --- |
| **Overview** \| **Package** \| Class \| **Tree** \| **Deprecated** \| **Index** \| **Help** \| **Weka's home** | |  |
| **PREV PACKAGE**   **NEXT PACKAGE** | **FRAMES**    **NO FRAMES**     **All Classes** |  |


---

## Package weka.classifiers.bayes

| **Class Summary** | |
| --- | --- |
| **AODE** | AODE achieves highly accurate classification by averaging over all of a small space of alternative naive-Bayes-like models that have weaker (and hence less detrimental) independence assumptions than naive Bayes. |
| **AODEsr** | AODEsr augments AODE with Subsumption Resolution.AODEsr detects specializations between two attribute values at classification time and deletes the generalization attribute value.  For more information, see:  Fei Zheng, Geoffrey I. |
| **BayesianLogisticRegression** | Implements Bayesian Logistic Regression for both Gaussian and Laplace Priors.    For more information, see    Alexander Genkin, David D. |
| **BayesNet** | Bayes Network learning using various search algorithms and quality measures.  Base class for a Bayes Network classifier. |
| **ComplementNaiveBayes** | Class for building and using a Complement class Naive Bayes classifier.    For more information see,     Jason D. |
| **DMNBtext** | Class for building and using a Discriminative Multinomial Naive Bayes classifier. |
| **HNB** | Contructs Hidden Naive Bayes classification model with high classification accuracy and AUC.    For more information refer to:    H. |
| **NaiveBayes** | Class for a Naive Bayes classifier using estimator classes. |
| **NaiveBayesMultinomial** | Class for building and using a multinomial Naive Bayes classifier. |
| **NaiveBayesMultinomialUpdateable** | Class for building and using a multinomial Naive Bayes classifier. |
| **NaiveBayesSimple** | Class for building and using a simple Naive Bayes classifier.Numeric attributes are modelled by a normal distribution.    For more information, see    Richard Duda, Peter Hart (1973). |
| **NaiveBayesUpdateable** | Class for a Naive Bayes classifier using estimator classes. |
| **WAODE** | WAODE contructs the model called Weightily Averaged One-Dependence Estimators.    For more information, see    L. |

---


|  |  |  |
| --- | --- | --- |
| **Overview** \| **Package** \| Class \| **Tree** \| **Deprecated** \| **Index** \| **Help** \| **Weka's home** | |  |
| **PREV PACKAGE**   **NEXT PACKAGE** | **FRAMES**    **NO FRAMES**     **All Classes** |  |


---
